# Through Their Eyes: Using Photovoice to Capture the Capacity‐Building Journey of Long Covid Patient Experts

**DOI:** 10.1111/hex.70094

**Published:** 2024-11-11

**Authors:** Nabil Natafgi, Katie Parris, Erin Walker, Tracey Gartner, Jeanette Coffin, Ariana Mitcham, Luis Sanchez Ferrer, Maushmi K. Patel, Haley Wymbs, Ann Blair Kennedy

**Affiliations:** ^1^ Department of Health Services, Policy, and Management University of South Carolina Columbia South Carolina USA; ^2^ University of South Carolina Patient Engagement Studio Greenville South Carolina USA; ^3^ University of South Carolina School of Medicine Greenville Greenville South Carolina USA; ^4^ Department of Biomedical Sciences University of South Carolina School of Medicine Greenville Greenville South Carolina USA; ^5^ Family Medicine Department Prisma Health Greenville South Carolina USA

**Keywords:** Long Covid, patient engagement, patient experience, photovoice, qualitative research

## Abstract

**Background:**

Long Covid, characterised by persistent symptoms following the coronavirus disease 2019 (COVID‐19) infection, significantly impacts the quality of life. Engaging patients in research and care through participatory methods can enhance a shared understanding of illness and improve the relevance of research.

**Objective:**

We define Patient Experts (PEs) as persons (including patients, caregivers and providers) who have completed a series of training sessions on team building, research methods and communication at the Patient Engagement Studio, University of South Carolina (PES USC). This study explores the use of photovoice to document the experiences and capacity‐building journey of Long Covid PEs within PES USC.

**Methods:**

The study employed photovoice within the COVID‐19‐Focused Virtual Patient Engagement Studio (CoVIP Studio). PEs submitted photographs and narratives at two distinct time points. Among the 18 PEs who participated in the project, 47 photos were collected during the training, and 31 were collected at the project's conclusion. Thematic analysis was conducted to capture changes in patient perspectives and engagement.

**Results:**

Initial themes identified were “Hope through Community,” “Collaborative Education and Research” and “Strength and Endurance.” By the project's end, themes had evolved to “Working as a Team to Share and Acquire Knowledge,” “Enhanced Confidence in the Future of Care” and “Perseverance and Progress.” These findings highlight the transformative impact of patient engagement and the utility of photovoice in documenting longitudinal shifts in patient perspectives.

**Conclusion:**

Photovoice effectively engaged Long Covid patients and captured their evolving roles and perceptions as PEs. The study underscores the value of patient‐led participatory methods in enhancing the relevance and applicability of clinical research, advocating for their broader adoption to improve patient‐centred care and research outcomes.

**Patient or Public Contribution:**

A CoVIP Studio stakeholder advisory board (CoVIP SAB) guided the co‐development and implementation of this project. The CoVIP SAB comprised nine members with complementary skills and expertise, including three patients, three clinicians who provide care to patients with COVID‐19 and three researchers with expertise in patient‐centred research, COVID‐19 and/or patient engagement and collaborate with patients as co‐investigators. The board contributed to project design and implementation, refining photovoice prompts and shaping dissemination strategies. In addition, one PE who actively participated in all phases of the project contributed to the writing of this paper and is a coauthor. All project activities involved patients and/or caregivers with lived experience of Long Covid.

## 1 Introduction

### 1.1 Patient Engagement in Long Covid Research

In response to the growing need for research focused on Long Covid, patient engagement has emerged as an important strategy for developing patient‐centred, innovative solutions [1, 2]. The Patient‐Centered Outcomes Research Institute (PCORI) defines patient engagement in research as “meaningful involvement of patients, caregivers, clinicians, and other healthcare stakeholders throughout the entire research process, from planning the study to conducting the study, and disseminating study results” [3]. Patient engagement emphasises the partnership between researchers, medical professionals and individuals affected by Long Covid [4, 5, 6]. An increasing prevalence of Long Covid and a lack of knowledge base on therapeutic options necessitated patient participation in research to understand better the experiences of individuals suffering from Long Covid [4, 5, 6]. Literature has used different terms like ‘patient partners’, ‘patient advisors’, ‘co‐researchers’, ‘patient research partners’ and so forth; for patients engaged in research, we use the term ‘Patient Experts’ [4, 7, 8, 9, 10]. We define Patient Experts (PEs) as persons (including patients, caregivers and providers) who have completed a series of training sessions on team building, research methods and communication at University of South Carolina (USC) Patient Engagement Studio (PES).

Training programmes that equip PEs with skills and knowledge and build the capacity to actively participate in research are essential for achieving meaningful patient participation [4, 8, 11, 12, 13, 14]. Involvement of PEs in research not only ensures the relevance and applicability of research questions and methods but also enhances patient‐centred care, ultimately improving health outcomes [2, 5, 14, 15, 16, 17]. However, the benefits of patient engagement and the evolution of the patient throughout the process have yet to be fully examined [7, 10, 11, 18, 19].

### 1.2 Photovoice

Photovoice, a qualitative research method, is a unique approach to capturing the lived experiences and perspectives of individuals in a visually compelling and participatory manner [20]. Researchers across various disciplines have employed photovoice as an effective engagement tool to enable the participants to express their feelings and concerns, share their perspectives and foster meaningful conversations among research participants [21, 22, 23, 24]. Online photovoice, a modified version of traditional photovoice developed during the pandemic, facilitated culturally and contextually appropriate, efficient and effective research engagement when face‐to‐face participation was not possible [24, 25]. In healthcare, the photovoice methodology facilitates a deeper understanding of the impact of disease on individual patients and communities through the lens of those experiencing and living with the disease [26, 27, 28, 29, 30, 31].

By allowing patients to document their experiences through photography, photovoice empowers participants to express their concerns, challenges and aspirations authentically [32]. It also gives voice to patients who may otherwise not be heard, including those who have language barriers or have trouble articulating their experience [27, 28, 30]. This visual participatory approach acknowledges patients as experts in their own lives, fostering a sense of ownership and agency in driving positive change within healthcare and research processes [20, 32, 33]. This methodology has been employed by researchers on a variety of conditions and patient populations, mostly at a single time point [27, 30, 34]. To our knowledge, photovoice has rarely been used as a tool for longitudinal evaluation of capacity‐building and patient engagement activities, especially within the context of Long Covid.

### 1.3 Objectives

The study explores the evolution of patients in a capacity‐building project using photovoice within the context of Long Covid research. Specifically, we examine how individuals with Long Covid, referred to as PEs, perceive and define their roles throughout their engagement in the coronavirus disease 2019 (COVID‐19)‐Focused Virtual Patient Engagement Studio (CoVIP Studio). Through an analysis of photovoice submissions and participant narratives at multiple time points, we aim to elucidate the impact of research training on capacity building among PEs. The proposition is that the training and the interactions with the researchers and other PEs will result in capacity building. Current literature on the use of photovoice in healthcare is restricted to understanding lived experiences, health education and participation assessment [26, 28, 30, 32, 33, 34, 35]. By shedding light on the evolving role of PEs and the utility of photovoice in patient engagement, this study contributes to a better understanding of collaborative research approaches in the context of Long Covid and lays the groundwork for future initiatives aimed at enhancing patient‐centred care and research.

## 2 Methods

### 2.1 Context

The PES unites a diverse group of patients, researchers, clinicians and community members with the mission of incorporating patient and community voices in all stages of research, thereby optimising both research processes and patient outcomes [5, 36]. PEs are patients and caregivers who can leverage their lived experiences with a particular condition or population group as expertise in assisting healthcare researchers and innovators [4, 5, 6, 14, 16, 36, 37].

#### 2.1.1 Theoretical Framework

Our study uses the grounded theory approach to unravel the capacity‐building journey through the experiences of PEs at the PES [38, 39, 40]. The theoretical perspective of symbolic interactionism associated with evolved grounded theory relies on the subjective meaning the participants place on their experiences and interactions [39, 41, 42]. Aligning with this perspective, we use photovoice to explore the capacity‐building journey of the PEs [39, 41].

### 2.2 Study Design, Setting and Project Overview

This study employs the qualitative methodology of photovoice to explore the experiences of PEs using a subset of data from a larger project that established the CoVIP Studio [5]. From fall 2021 to spring 2023, a nationwide network was developed, training individuals experiencing Long Covid, including patients, caregivers and clinicians, to become PEs. These PEs, from 17 states, provided valuable lived experiences and feedback throughout the project to enhance Long Covid research. Over 5 months, eight training sessions were conducted, incorporating homework assignments, team‐building activities and various learning topics. Once training was completed, PEs worked as a team to complete 20 research review sessions with researchers from more than 15 institutions. The end of the project was celebrated with a final convening meeting with the PEs, members of the advisory board and researchers.

### 2.3 Reflexivity

The project team encompasses an array of experience and expertise, including PEs, Stakeholder Advisory Board (SAB) members and the PES team including faculty, staff and students (medical, masters level and doctoral level). Of note, nearly all the PEs contracted COVID‐19 in the early days of the pandemic when vaccines were not yet available (before June 2020) and had been experiencing Long Covid symptoms for over a year. All but one of the project staff members had also contracted COVID‐19 at least once, and at least one suspects they may also be experiencing mild Long Covid symptoms. All members of the team, including project staff and PEs, are living with the implications and outcomes of Long Covid for both them and those they live, work and play with.

The intersection of lived experience with Long Covid among the PEs and their role in the research process enriched our collective insights and contributed to a more nuanced understanding of the data. However, it is crucial to recognise that this overlap also introduced complexities in interpretation. Our personal journeys with COVID‐19, particularly during the early, uncertain days of the pandemic, could have predisposed us to identify more strongly with themes of resilience and community support. To counteract this potential bias, we employed facilitated discussion sessions, ensuring all researchers and PEs alike had equal opportunities to influence interpretations. Moreover, we incorporated iterative feedback loops, where preliminary findings were revisited with PEs to refine our themes and ensure they accurately reflected the varied experiences within the group. These strategies not only enhanced the inclusivity and depth of our analysis but also helped safeguard the integrity of our findings, allowing us to navigate the complexities introduced by our personal connections to the research subject.

### 2.4 Data Collection

Data was collected at two distinct time points. Phase One occurred during the training sessions; during the fifth training session, patients were provided with homework and were asked to provide three to five pictures answering the prompt: “*Upon completing your training you will be considered a “patient expert,” what does that look like for you?*”. Photos were submitted using a Slack channel in which all participants could observe, interact and comment on others' photos and posts. All activities associated with the photovoice activity were conducted virtually. All photos were then gathered into a PowerPoint presentation with a slide created for each participant to be presented during the next training session. Each participant presented their slide and further explained why their photos represented their idea of becoming a PE. This meeting was recorded and transcribed.

Phase Two data collection occurred at the conclusion of the CoVIP project, where the now PEs were asked to provide three to five photos, addressing the prompt: “What does it mean to be a Patient Expert?”. Photos for this round were submitted via email or text message and compiled into the final PowerPoint presented at the convening meeting. The Slack channel used to collect data for Phase One was found to be cumbersome by some of the PEs, so alternative options of email and text were provided for submitting photos for Phase Two. PEs were then invited to share the context and significance behind their photos, either orally during a virtual meeting or via writing submitted to the Program Manager. All photos, photo descriptions, meeting transcripts and meeting notes were compiled for analysis.

### 2.5 Data Analysis

Based on the grounded theory, the data analysis was an iterative and bidirectional process between the researchers and the PEs. Researchers conducted a thematic analysis of the collected photos, descriptions, meeting transcripts and meeting notes at the end of each phase of data collection in a group. Collaborative qualitative data analysis has been reported to enhance the rigour and quality of research, especially in cases where research teams include interdisciplinary researchers, academics and practitioners, patients, students and novice researchers [43, 44]. Peer debriefing and researcher triangulation are incorporated into the collaborative data analysis process, thereby increasing the credibility of the results [44].

During Phase One exercises, PEs submitted photos in response to the prompt “Upon completing your training you will be considered a “patient expert,” what does that look like for you?”. Open coding was the first step in data analysis after the photos were received. Researchers identified codes from the data shared by the patients. Researchers then categorised the codes into seven themes to demonstrate the commonalities in the data shared by individual PEs. In the next step, the themes were presented to the PEs in a virtual meeting. Member‐checking procedures were performed by asking how well the findings represented their experience and whether there were any misinterpretations. Axial coding was performed next with input from PEs on how to form connections between the seven themes, combine them into fewer themes and identify subthemes under each theme.

After the training, Phase Two of our study, PEs submitted photos in response to the question, “What does it mean to be a Patient Expert?”. Open coding procedures were performed as in Phase One and themes were created from the codes identified. Axial coding was performed with input from PEs along with the member‐checking procedure similar to Phase One. Finally, the researchers presented the combined themes and identified subthemes from Phase One and Phase Two to the PEs and asked the PEs' perspectives on how the themes had evolved from Phase One to Phase Two. Further analysis was conducted to consolidate the information shared by the PEs to identify the trends and shifts in their role.

PEs did not have any major criticism of the themes provided and many commented that they felt the themes did accurately encompass their experience as a PE. One comment was that they felt the concept of feeling as a vital member of the research team was not fully captured in the initial findings. This was addressed by changing the theme of “Unclouding the Research Process” to “Vital Members of the Research Team.” Furthermore, the definition of perseverance and progress was expanded to demonstrate capacity building among the PEs who were inspired to expand their role and get further involved in research and advocacy on other health conditions in addition to Long Covid.

The collaborative data analysis followed a dynamic approach where not only did researchers work together in a group but all PEs actively engaged in a collaborative discussion on member‐checking procedures and axial coding procedures. As noted in the literature, this allowed us to develop consistency, agreement and transparency in data analysis without calculating intercoder reliability [44]. We describe the trustworthiness of the data analysis procedure in the following section.

### 2.6 Trustworthiness

Lincoln and Guba [45] have referred to research quality and reliability in qualitative research as trustworthiness. In alignment with Lincoln and Guba's [45] criteria for assessing the trustworthiness of qualitative research, we implemented several strategies to ensure the credibility, transferability, dependability and confirmability of our findings. Member‐checking procedures were instituted to establish credibility and ‘truth of findings’ by allowing participants the opportunity to review and validate aspects of the interpretations of the data they provided [46]. A summary of the results was shared with the PEs during a synchronous virtual session and feedback on accuracy and validity was acquired. Through facilitated discussions, we explored how participants' perspectives and priorities may have shifted or evolved in response to ongoing engagement in the research process. This reflexive approach enabled us to refine and revise our interpretations considering participant feedback, thereby enhancing the trustworthiness and authenticity of our findings.

Detailed descriptions of our methodology, quotes, context and participants were shared via disseminated materials to enhance transferability. The SAB members played a crucial role throughout the project, preserving dependability. SAB members examined the processes and products of the project, and they also evaluated the findings, interpretations and conclusions. Research triangulation, an inherent feature of collaborative data analysis, ensured confirmability [44]. The researchers and PEs reviewed the codes and themes together, and transparency and agreement were established during the data analysis procedures, thereby reducing individual bias and enhancing the reliability of our interpretations.

## 3 Results

### 3.1 CoVIP Panel Membership

The CoVIP Panel for this project is composed of a diverse group of individuals, including those with Long Covid, caregivers and clinicians. The panel includes a majority of women and represents a variety of racial identities and educational backgrounds, ranging from some college experience to advanced degrees. Members of the PES span different age groups, gender and racial/ethnic identities and geographic regions across the United States, bringing a wealth of perspectives from various urban and rural settings. This diversity enriches the research by incorporating a broad spectrum of experiences and insights.

The majority of our PEs for this project were females, White and in the age group of 30–49 years. We do not include detailed demographics of our PEs for several reasons. First, our aim is not to generalise the findings but to ensure representation, which is particularly relevant in qualitative research where the richness of individual experiences is paramount. Second, due to the small size of our group, providing specific demographic details could inadvertently lead to the identification of individuals, especially within certain intersectionalities. Additionally, our approach recognises PEs as research consultants rather than mere data points, emphasising their contributions and insights over their demographic profiles. This perspective helps respect their privacy, aligning with ethical standards in participatory research. Finally, focusing on the collective narrative and shared experiences of the group enhances the depth and relevance of our findings, which are intended to inform and improve patient‐centred care and research outcomes.

### 3.2 Phase One Findings

**Figure 1:**
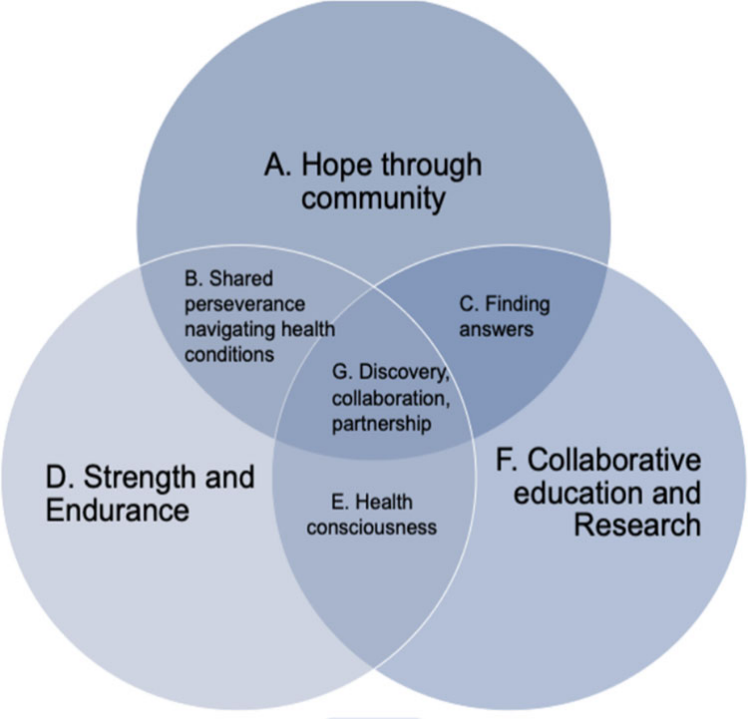
Graphic representation of overlapping themes from Phase One.

Thirteen of eighteen PEs submitted 47 photos in response to the prompt, “Upon completing your training you will be considered a “patient expert,” what does that look like for you?”. In Phase One of the photovoice activity, several themes emerged from the contributions of the PEs as they documented their experiences with Long Covid. The seven themes identified were—hope, strength, collaboration, community, discovery, endurance and health consciousness. These themes encapsulated the shared challenges, strengths and community spirit that characterised their journey (Figure 1).

Following validation of the codes identified and the themes created, final categories of themes and subthemes were created based on the input from the PEs. The final themes were: “Hope through Community”, “Strength and Endurance” and “Collaborative Education and Research”. The subthemes that were identified were: “Shared Perseverance Navigating Health Conditions”, “Discovery, Collaboration, Partnership” and “Finding Answers”.“*Hope through community*” was defined as the ability to find solace during uncertainty thanks to the support of their community. The team defined “*Strength and Endurance*” as feeling empowered to navigate through a difficult time for themselves and many others. Finally, “*Collaborative Education and Research*” was defined as the cumulative efforts required to be able to provide insights into scientific research from the patient perspective. Table 1 highlights the themes and overlapping sub‐themes with supporting quotes and photos to represent the concepts identified by the team.

**Table 1:** Themes and illustrative quotes from the Phase One photovoice exercises.

Theme	Description	Illustrative image	Illustrative quote
A. Hope through community	Many Patient Experts (PEs) emphasised the importance of community support in coping with Long Covid. This theme highlighted the collaborative efforts and emotional sustenance provided by networks of friends, family and fellow sufferers.	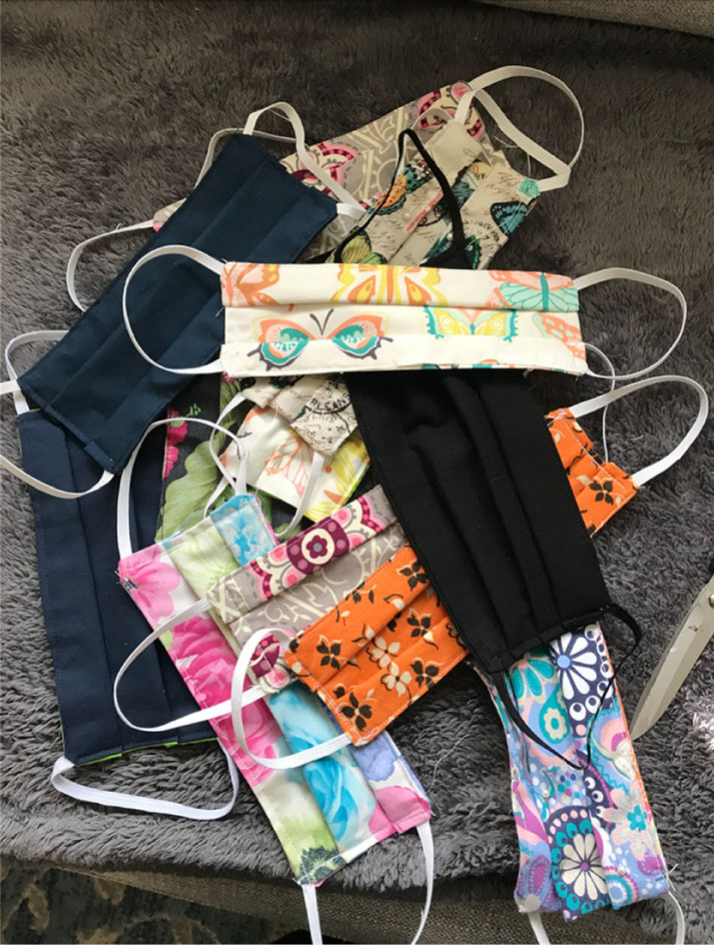	“These are some of the hundreds of cloth masks I sewed early on in the pandemic. I got sick in March 2020. While sick, I watched the news, reporting on numbers of people dying. It was a frightening time for all. I have friends and family who are essential workers and they were unable to get masks. I was too sick to work but after a few weeks, I knew I could sit in front of my sewing machine. In between naps and nebuliser treatments, I sewed. Everyone was asking for masks. When I ran out of fabric and elastic, friends and family sent me whatever they had on hand, raiding their moms and grandmothers sewing supplies. Soon I found a FB group of community members who were also sewing. One wonderful woman organised us and many people stepped in to help. If they didn't sew, they cut fabric, or donated thread, or picked up other supplies. A metal working shop fabricated nose pieces. A network of unseen volunteers were delivery personnel and dropped off or picked up what we needed on our doorsteps. Orders came in and I just sewed. Instead of feeling sick and depressed, I had a purpose. Our Patient Engagement Studio reminds me of this. A group of people, coming together for a purpose. We are learning together. Everyone has something to contribute.”
B. Shared perseverance navigating health conditions	This theme focused on the collective struggle and resilience of the PEs as they dealt with the persistent and often baffling symptoms of Long Covid. It showcased their determination to adapt and find ways to manage their new realities.	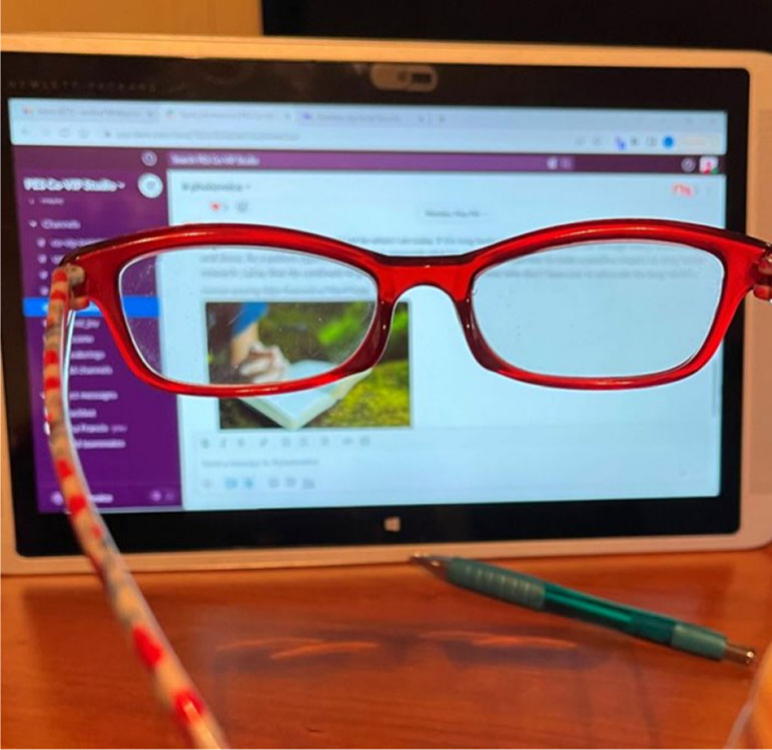	“The first round of Covid brought with it lots of new physical challenges, most defying diagnosis or explanation. The second round only exacerbated many of those issues. One of the more challenging of my personal “new normals” has been vision difficulties.” “Pre‐Covid my vision was better than 20/20. Post‐Covid eye exams have shown 20/20 vision, but prolonged periods of unexplained blurry vision is a new reality—and now so are reading glasses. Participating in the CoVIP project gives me hope that there will soon be a greater understanding of some of the more baffling aspects of Long Covid for those left struggling in its wake with no answers. …and then there's the added benefit of building a collection of really cute reading glasses.”
C. Finding answers	The quest for understanding and effective treatment was a recurrent theme. PEs expressed their frustration with the medical system and the need to seek answers independently or through alternative channels.	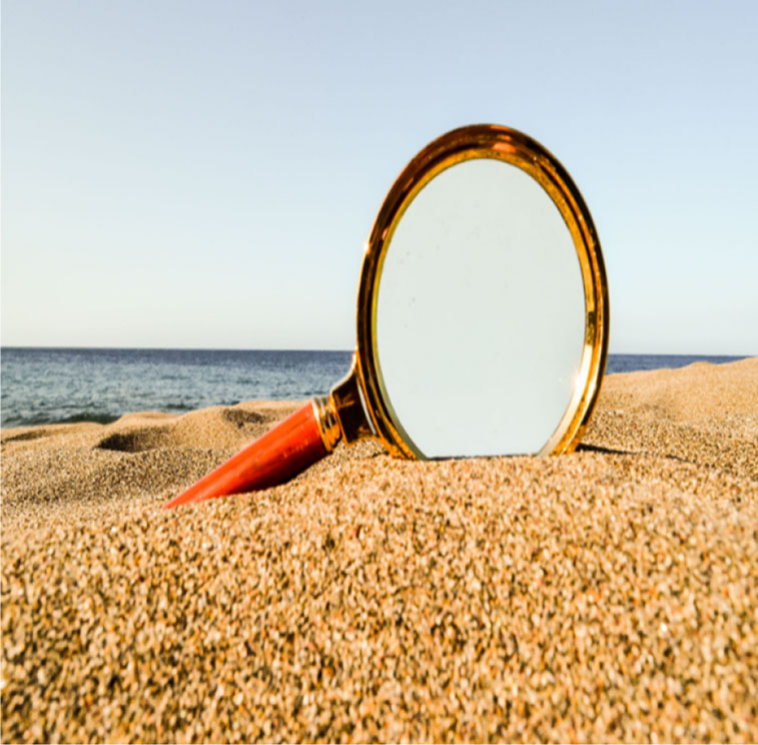	“I have to search for answers myself, because my doctor, I've just given up on him. He really has no interest in trying to help me figure out what it is that can help me. I really feel like I'm on my own. But at the same time, at least I have some others to talk to that are outside of my doctor's office. But it's really just communication… communicating and working together with people.”
D. Strength and endurance	PEs highlighted their internal strength and the sense of empowerment gained through participation in the engagement studio and contributing to research. This theme captured their resolve to persist despite ongoing challenges.	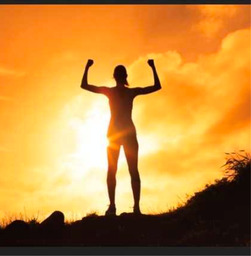	“During the past 18 months, I have felt powerless, desperate and at times hopeless because no one could help me. This photo to me demonstrates strength and empowerment. As a patient expert, I believe I will feel empowered because I am able to be a part of research that will hopefully help in my healing as well as the healing of thousands of other people suffering through the same thing. Being a part of positive change alongside others is such a great feeling of strength”
E. Health consciousness	The meticulous attention to health and self‐monitoring was a common thread. PEs described how they meticulously tracked their health parameters and adopted various health practices to manage their condition.	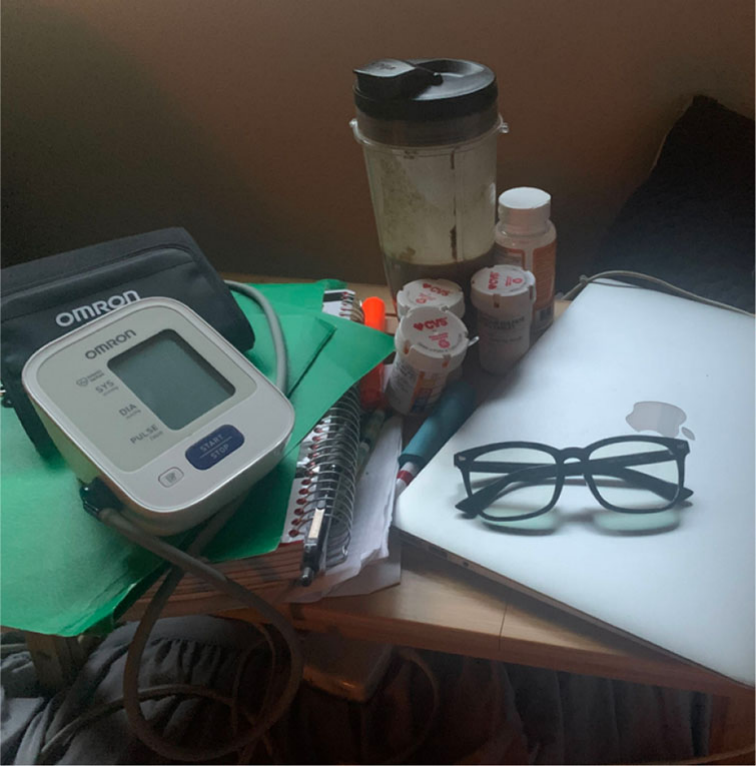	“[this picture] is all of my things that basically are by my bed, um, that I spent a lot of time on you know. Like glasses to help with the screen, my medications, tools to help me read, blood pressure cuff, all the things. And it's also speaking to the fact that, like, I feel like I'm a science experiment in real time and so by tracking everything going on with my body. It helps for the overall point of usage research.”
F. Collaborative education and research	The theme underscored the collective effort in learning and contributing to research. PEs valued the educational aspect of the engagement studio and felt their contributions were integral to advancing understanding of Long Covid.	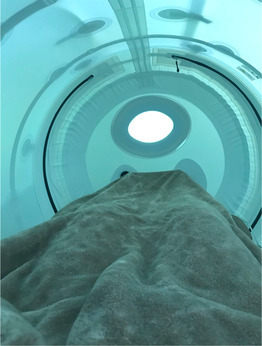	“We're pioneering our way through the next steps to see. What can we do and trying to share our journey to see if it can help others?”
G. Discovery, collaboration, partnership	This theme highlighted the joy and fulfilment found in collaborative discovery and partnership within the engagement studio. PEs felt a deep connection to the group's mission and the progress being made.	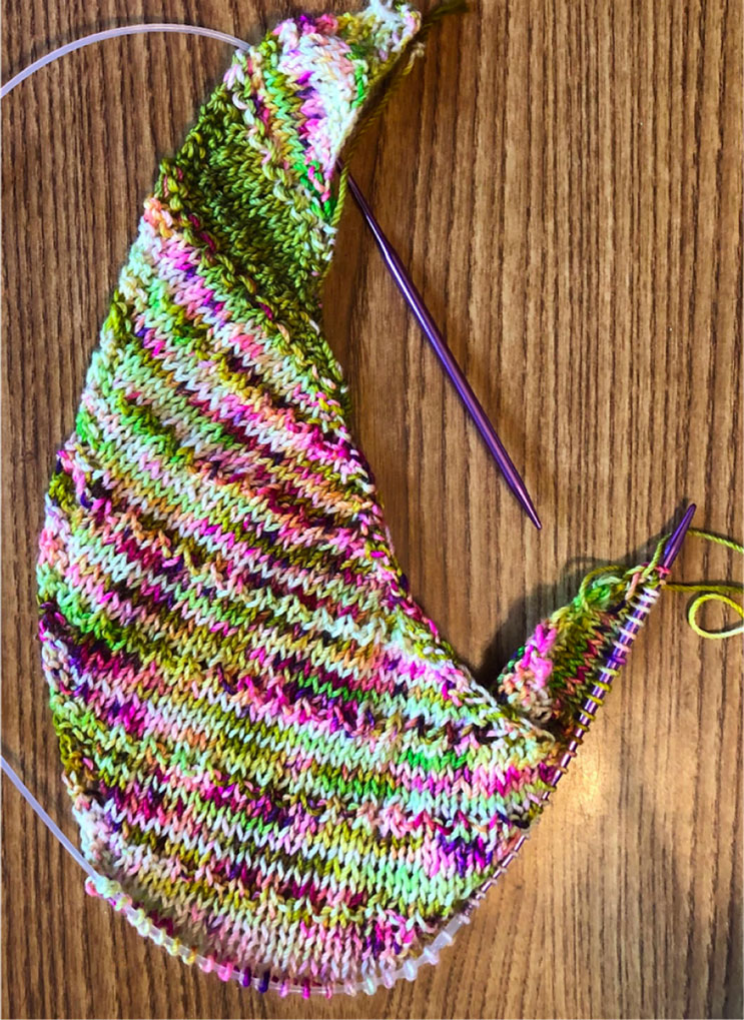	“When we had our first meeting together, I mentioned that I had been too busy during Covid to knit. I picked it up again recently and taught myself a new technique/slight correction. When I knit, I think, release, and pray. I was thinking of how much I learn from the people in this group and in general about Covid patients. There is dark and light in this knitted piece, just like the battle we wage against Covid. And the pandemic has woven so many of us together in ways we never anticipated. As knitting goes, it takes shape as the rows are finished and becomes a beautiful piece. I feel confident that the same will be true as we move forward with the Patient Engagement Studio.”

### 3.3 Phase Two Findings

**Figure 2:**
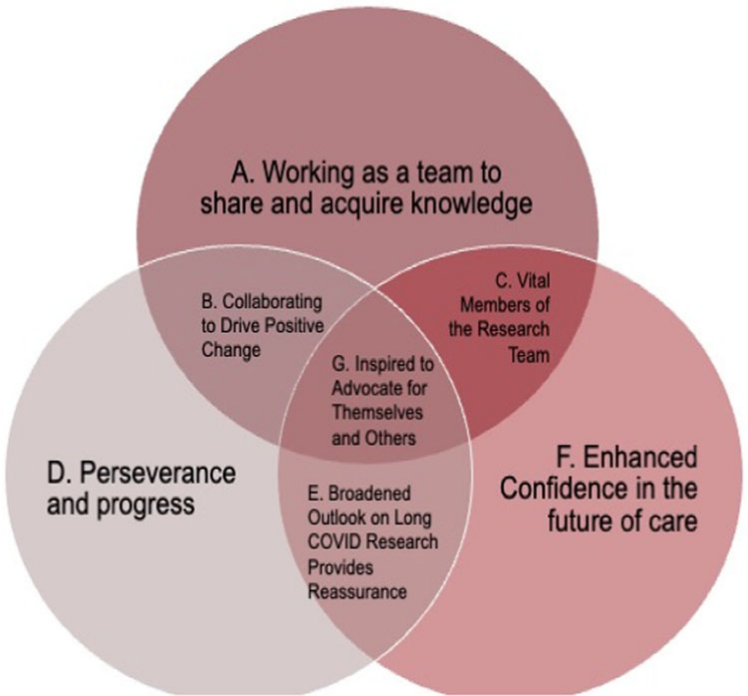
Graphic representation of overlapping themes from Phase Two.

In response to the prompt, “What does it mean to be a Patient Expert?”, 11 PEs submitted 31 photos for Phase Two. The initial themes identified were surrounding—knowledge, cooperation, victory, growth, partnership, expanding scientific knowledge, further research, sharing experiences, advocacy, optimism about the future, community and hope. Thematic analysis once again produced three overarching themes; “Working as a Team to Share and Acquire Knowledge”, “Enhanced Confidence in the Future of Care” and “Perseverance and Progress” (Figure 2). Thematic analysis findings from Phase Two revealed sub‐themes—“Collaborating to Drive Positive Change”, “Inspired to Advocate for Themselves and Others” and “Vital Members of the Research Team”, which fit multiple overarching themes (Table 2).

**Table 2:** Themes and illustrative quotes from the Phase Two photovoice exercises.

Theme	Description	Illustrative image	Illustrative quote
A. Working as a team to share and acquire knowledge	Patient Experts (PEs) highlighted the value of collaborative learning and sharing knowledge within the team.	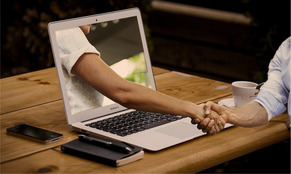	“After every session, I felt that I learned so much. Not only from the presenting researcher, but from the whole team as well. I often researched the topic beforehand if I could, and after the session as well. I wanted to represent the knowledge I gained from the researcher as well as the sharing of ideas with everyone on the call.”
B. Collaborating to drive positive change	PEs emphasised the importance of interconnectedness and the potential for their collaborative efforts to create positive future outcomes.	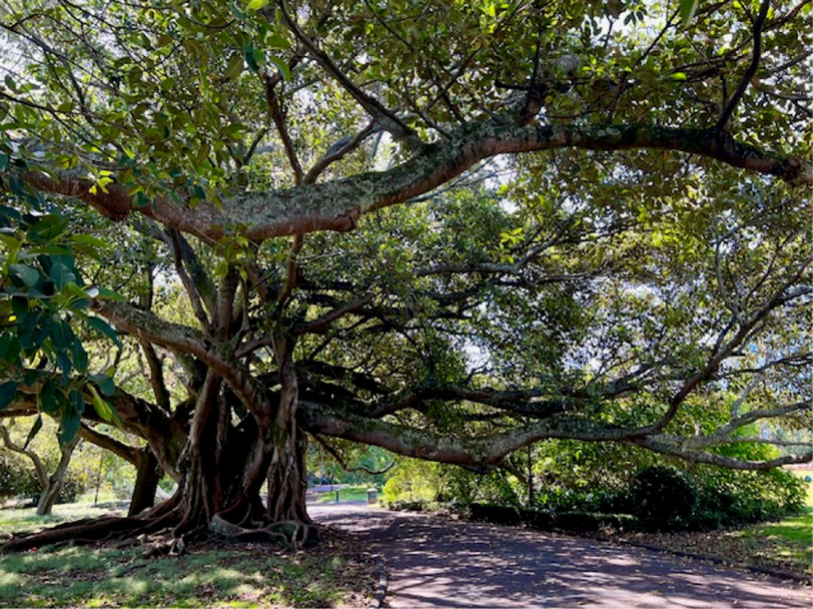	“[the tree] represents for me, not only our interconnectedness here, but that these branches we've created will create good wonderful things and more connections in the future.”
C. Vital members of the Research Team	PEs expressed pride and excitement about their active and integral roles in research projects.	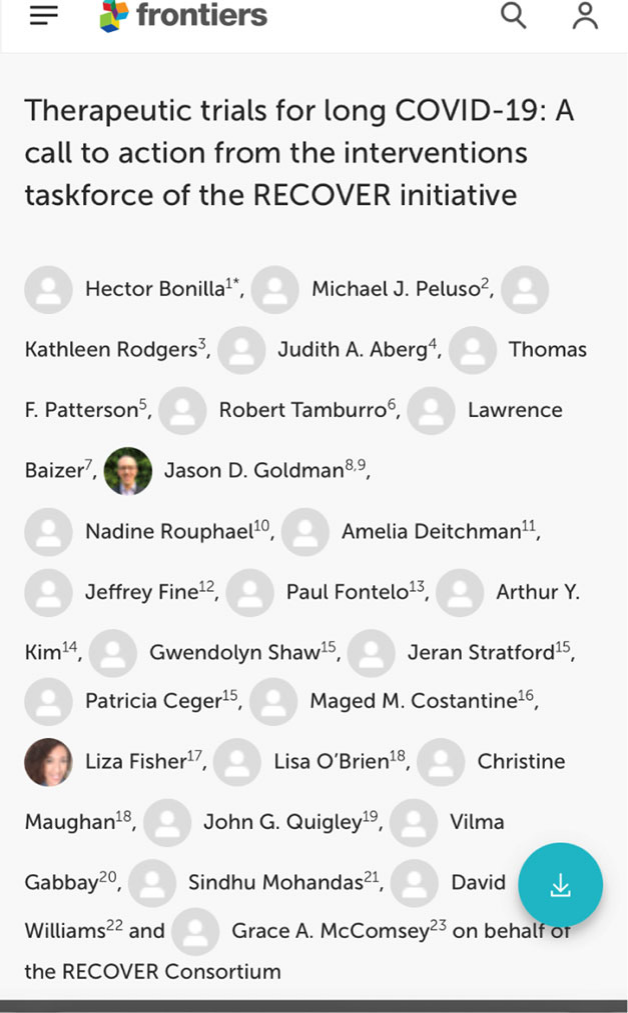	“I had my second research manuscript get published that I was able to co‐author on and so that's like something really important to me and kind of excited about that. So I've been able to take some of these trainings and some others and work in some other spaces, including the NIH, and contributing and doing some patient input in the clinical trials.”
D. Perseverance and progress	PEs felt a sense of growth and progress, both personally and within their roles as patient experts.	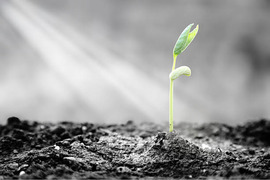	“The 1st picture with the sprout and the sunlight to me, like, it just seems like with the right light or for us, like the right opportunity, there's new growth as a patient expert. I just feel like we have grown as we've been given this new opportunity to share our experiences, and assist the researchers.”
E. Broadened outlook on Long Covid research provides reassurance	Participation in the group expanded their understanding and provided reassurance about the ongoing research on Long Covid.	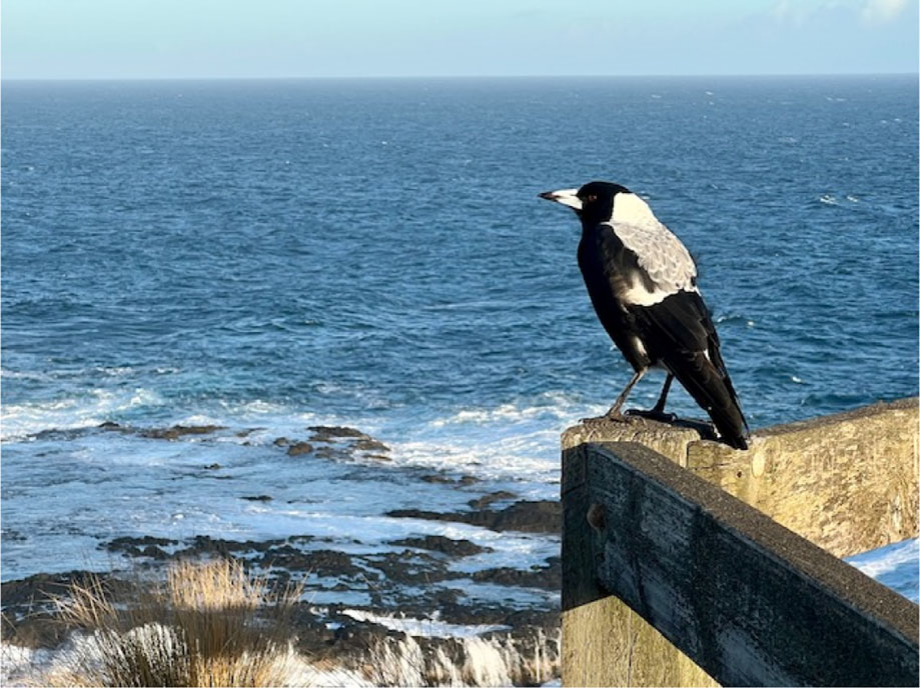	“I have a bigger outlook, and a bigger view about what's happening around, Long Covid research in particular because of our group.” “I just feel like things have really expanded and it's like, kind of has calmed me down and given me a lot of hope.”
F. Enhances confidence in the future of care	PEs developed greater confidence in the future of healthcare and the efforts being made to improve patient outcomes.	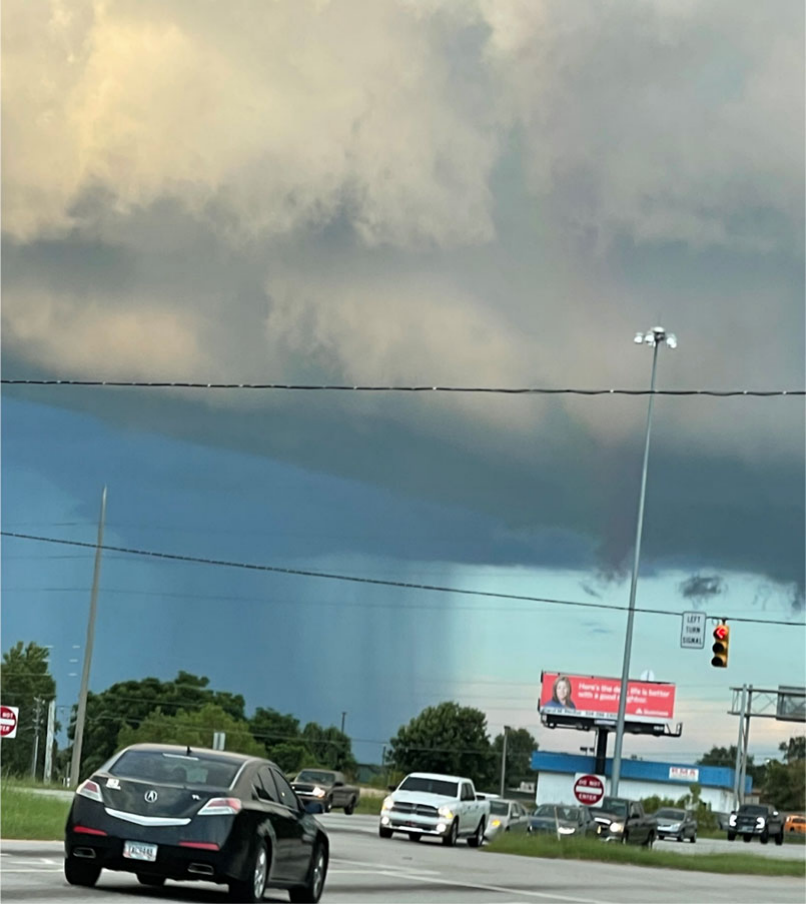	“I took this photo when driving home one afternoon and my route happened to be taking me directly into that storm. The stark contrast between the storm and the calm spoke to me because it was where I had been living for quite some time; stuck between the before and after of my trauma and illness. It remains challenging for me to find adequate treatment/diagnoses, but because of my experience with the PES I now know there are medical professionals who are working hard at helping patients figure out how to lessen the contrast between their before and after.”
G. Inspired to advocate for themselves and others	Participation in the program empowered PEs to take on advocacy roles and contribute to research initiatives.	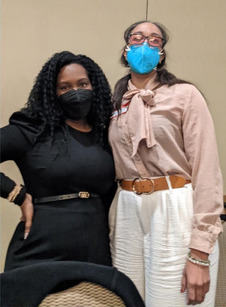	“I really wanted to get involved in research and I didn't know how…. as some of you guys know me and Liza launched our organisation that specialises in research in the middle of this, and we couldn't have done it, I would not have had the confidence to say 1. I am a patient expert, and then I wouldn't have had the experience already to be, like well, we've already worked with researchers. So now there's the reason why we shouldn't do more. So, this program really was integral in launching this [organization].”

### 3.4 Evolution of the PE Journey

**Figure 3:**
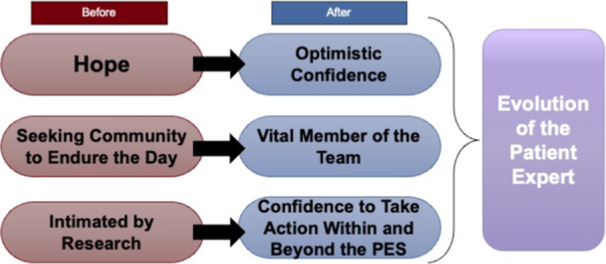
Thematic phase transition. PEs, Patient Experts.

The themes and subthemes that emerged from the photos collected at the end of Phase One and Phase Two demonstrated the capacity‐building journey of the PEs (Figure 3). Regarding the changes observed from the initial to final themes, a major transition was the shift from hope to optimistic confidence. Many PEs got involved with the PES to make a difference in the realm of Long Covid research but had less confidence that their efforts would result in meaningful change. However, through their time as PEs, they discovered that many researchers cared about them and were passionately working to find a way to improve the quality of life for those living with Long Covid. Actively working in driving the research forward further enhanced confidence and optimism among the PEs.

*As one PE shared*, “I was at a particularly low point and that rainbow appeared, and then disappeared within about a five‐minute time span. I felt as though it was a message from God, especially for me. I think he wanted to show me that even at my lowest points and hardest places there's always hope. This photo speaks to the hope that being part of the CoVIP panel has given me.”

Another shift that the PEs noticed was a movement from endurance to progress. They started as PEs just trying to make it through the day; however, by the end of the project, they were equipped and inspired to take action to combat their illness. Additionally, many gained a sense of confidence to push beyond their roles as PEs and use their newfound knowledge to get involved in research and patient advocacy on other conditions and outside of the PES.

*One PE noted*, “Being able to collaborate with professionals in the research space and co‐authoring manuscripts. Progress and hopefully treatments. Impacting what interventions are studied and made available. Being able to advocate not only for myself but for my community and those voices of the suppressed. The ability to communicate science clearly to the communities affected. Providing knowledge so others can benefit and feel empowered.”

Finally, another transformation noted was the transition from being a part of a community to a part of a team. They joined the PES to be surrounded by people who shared their ailment and thus feel less alone. However, throughout their time they became united not only by their disease but by working together to achieve a common goal. Their team was also not just the PEs themselves but also included the researchers who presented their projects to the group. Many PEs commented that a major transition they observed over their course of being a PE was becoming a valuable member of the research team.

*As mentioned by one of the PEs*, “I looked for a photo that represents the cooperation and teamwork both within the PES studio and with the researchers. It was very rewarding to see how the researchers valued our input.”

## 4 Discussion

In this project, visual narratives were used to capture the profound and nuanced transformations experienced by PEs with Long Covid. Thematic analysis of both phases of the project revealed a shift from feelings of isolation and desperation to a sense of communal empowerment over time. This transition highlights the crucial role of patient‐led research in addressing complex chronic conditions by capturing the lived experiences of the people that are frequently overlooked or underappreciated and thereby helping in tailoring patient‐centred disease management strategies [30]. Specifically, the use of photovoice methodology illuminates patient experiences that might otherwise remain private, addressing the well‐documented challenge of capturing the full patient experience [1, 29, 30, 35, 47].

### 4.1 Long Covid Research Development

The findings from this project align with existing literature on the benefits of engaging patients in research, especially those with chronic conditions [48, 49]. Many PEs, still experiencing the physical and mental effects of Long Covid, found the exercise powerful for multiple reasons. The symptoms of Long Covid vary from person to person and change over time in a person too [1, 50, 51]. Lack of acknowledgement that Long Covid is an illness by doctors and loved ones further complicated care access for patients. Photovoice provided a platform for Long Covid patients to discuss their symptoms and share their experiences [20, 30, 35, 50]. This process both humanised and validated their individual experiences, consistent with findings from other studies that emphasise the importance of patient validation in chronic illness research [27, 52, 53]. Many patients felt unable to discuss their feelings with others who may not understand the impact of Long Covid on their lives. Incorporating the Photovoice methodology enhanced the PEs' ability to build community, share their perspectives and feel heard [27, 54].

### 4.2 Photovoice as a Virtual Engagement Tool

While photovoice activities have been traditionally facilitated in person, this study was conducted virtually [31, 55]. This approach aligns with recent trends in leveraging digital tools for patient engagement, particularly in response to the COVID‐19 pandemic [5, 17, 56, 57]. Two strengths of this virtual engagement were identified. First, the study was able to be conducted at a reduced cost. In‐person photovoice often entails printing materials for both small group discussions and the final composition of thematic analysis, thus raising costs. Alternatively, the virtual format required the investment of time and effort but incurred minimal material costs [24, 25]. Second, the virtual format allowed for an increased diversity of reachable patients [24, 25].

The virtual format allowed for participation by PEs who may have otherwise been unable to attend due to physical limitations, including fatigue experienced due to Long Covid. This finding is supported by literature highlighting the benefits of virtual engagement in overcoming geographical and physical barriers [17, 24, 49, 57, 58]. It also excluded travel time as a barrier to participation, allowing the inclusion of PEs who are geographically dispersed. Photovoice tailored to a contextually appropriate setting like Long Covid resulted in the collection of rich and high‐quality data where conventional qualitative data collection methods would not have been appropriate or feasible for the participants as noted by researchers in different settings [24, 29, 30, 32, 33, 34, 35, 50]. The data collected not only provided insight into the thoughts of patients at the beginning of their journey but also highlighted the transformation experience while becoming a PE.

### 4.3 The Evolution of the PE

Photovoice proved beneficial for team building. At the beginning of the project, PEs reported that photovoice was a helpful tool for facilitating introductions and establishing rapport among members of the PE team. This aligns with existing studies on the use of photovoice in enhancing group cohesion and trust [59, 60]. Photovoice was also useful for evaluating PE experiences and personal growth. While there is a gap in the literature regarding the evolution of PE experience, our study revealed a marked shift from feelings of isolation and disconnection with research to a strengthened communal and empowered stance. At the conclusion of the project, PEs revealed that photovoice was a beneficial tool in allowing them to reflect upon their experience and contemplate the personal development they gained from their participation. Photovoice as a method may, therefore, assist in understanding how patients' perceptions have grown over time, providing a valuable tool for longitudinal studies on patient engagement [61].

As research emerged and the body of knowledge regarding COVID‐19 evolved, patients became frustrated with the changes in recommendations and guidelines. Meeting with researchers allowed PEs to gain a better understanding of the difficulties associated with conducting medical‐related research and dispelled some of this frustration. This is consistent with other studies that emphasise the importance of patient education in mitigating frustration and enhancing engagement [58]. Throughout the life of the project, PEs had the opportunity to provide feedback and insight to researchers. This was particularly impactful for the PEs who were unable to return to work due to limitations caused by Long Covid, as both this exercise and participation in the PES overall provided a sense of purpose. For many PEs, the feeling of being on equal footing with highly educated and experienced researchers was a unique and uplifting experience as well. This is consistent with the results of other studies focused on photovoice as an engagement tool [6].

### 4.4 Limitations

While this study provides valuable insights into the experiences and evolution of PEs with Long Covid, it is not without limitations. The relatively small and specific sample of PEs with Long Covid limits the generalisability of the findings to broader populations, including those with different conditions. The voluntary nature of participation may have introduced selection bias. Those who chose to participate might have been more motivated or had more positive attitudes towards engagement in research than the broader population of Long Covid patients. Some of the participants who agreed to participate in the study did not submit photos (five in Phase One and seven in Phase Two). However, it was noted that even the participants who did not submit the photographs participated actively in the discussion on themes and subthemes, thereby contributing to the analysis procedures.

Furthermore, the interpretation of photographs and narratives is inherently subjective. Although the inherent researcher triangulation incorporated within collaborative data analysis, the member checking procedures performed during the analysis phase and reflexive discussions mitigate the researcher bias, the personal experiences of the researchers as well as PEs could still influence the findings. The study included PEs who were already involved in virtual engagement as a part of a larger CoVIP project. Therefore, the participants had no difficulties in access and use of technology. This made it difficult to address the digital barriers that might be prevalent in Long Covid patients not included in our study. Lastly, the ability of PEs to participate and complete the Photovoice activity was dependent on their health status. Those who were extremely debilitated by Long Covid symptoms were unable to participate, potentially skewing the findings towards those with milder symptoms.

### 4.5 Implications for Future Research

As indicated in our study, photovoice was found to be contextually appropriate for Long Covid patients by the researchers, and the PEs also found it beneficial. Online photovoice was developed by Tanhan and Strack [24] to make traditional photovoice more culturally and contextually appropriate so that participants can express their experiences. Future studies could leverage the use of photovoice as an effective tool to capture the lived experiences of the participants. Furthermore, Tanhan [25] also developed Online Interpretative Phenomenological Analysis (OIPA) as an analysis tool that could be used effectively with photovoice methodology. This study calls for future qualitative and mixed methods research to incorporate OIPA with the photovoice methodology for the generation of an effective evidence base.

Our study explored the potential of photovoice as a methodology in team building among participants, providing a platform to share their experiences with Long Covid, and understanding the capacity‐building journey while becoming a PE. Literature shows evidence of photovoice in researcher training and assessment of community partnerships [32, 35]. Photovoice with OIPA could be leveraged to measure the level of engagement and quality of engagement in Patient‐Centred Outcomes Research and Community Based Participatory Research.

We investigated the capacity‐building journey of the PEs; future studies could explore specific areas like mental health struggles and health‐related quality of life of patients with Long Covid. Another implication of photovoice could be understanding the lived experiences of people with chronic diseases like diabetes and hypertension. Photovoice will aid in capturing the individual perspectives and complex struggles of persons living with chronic conditions that are otherwise unappreciated in clinical settings that limit the interactions between providers and patients [30]. This could be beneficial in designing patient‐centred treatments for people with chronic conditions and empower patients for self‐management.

## 5 Conclusions

Photovoice is a viable tool for virtual participatory research and patient engagement in research. It illuminates the subjective, and oftentimes marginalised, experiences of PEs and fosters an inclusive, collaborative research environment. This study utilised photovoice to facilitate a space where PEs could visually and narratively share their journeys. This approach has underscored the value of the patient's lived experience in shaping research agendas and has broad implications for enhancing patient‐centred research methodologies across various health conditions, advocating for a model that is adaptable, cost‐effective and profoundly impactful.

## Author Contributions

**Nabil Natafgi:** conceptualisation, methodology, investigation, supervision, resources, funding acquisition, writing–original draft, writing–review and editing. **Katie Parris:** project administration, writing–original draft, writing–review and editing, data curation, formal analysis. **Erin Walker:** formal analysis, data curation, writing–review and editing, writing–original draft. **Tracey Gartner:** formal analysis, writing–review and editing, data curation, writing–original draft. **Jeanette Coffin:** writing–review and editing, validation, writing–original draft. **Ariana Mitcham:** writing–review and editing, data curation, writing–original draft. **Luis Sanchez Ferrer:** writing–review and editing, writing–original draft. **Maushmi K Patel:** writing–review and editing, writing–original draft. **Haley Wymbs:** writing–review and editing, writing–original draft. **Ann Blair Kennedy:** conceptualisation, methodology, supervision, validation, writing–original draft, writing–review and editing, funding acquisition, resources.

## Ethics Statement

This project was approved by the University of South Carolina Institutional Review Board (IRB) for the PES (Protocol ID: Pro00129024). All project activities, including the implementation of the Photovoice methodology, adhered to the ethical guidelines and protocols established by the IRB to ensure the protection of participants' rights, privacy and confidentiality.

## Consent

All participants provided informed consent before participating in the CoVIP Studio activities.

## Conflicts of Interest

Natafgi and Kennedy received multiple funding from PCORI. Natafgi and Kennedy also received honoraria from some institutions related to their consultancy work on methodologies of engaging patients in research, none of which are directly related to this work. Coffin has been financially compensated for her time as a Patient Expert on this project. Parris is an employee of the PES. Walker, Mitcham and Ferrer received financial compensation from the PES as graduate research assistants. All authors had access to data and contributed to the writing of the manuscript.

## Data Availability

Since this was an engagement project and not a research project, data are available on reasonable request due to privacy/ethical considerations.
